# Refining Black men’s depression measurement using participatory approaches: a concept mapping study

**DOI:** 10.1186/s12889-021-11137-5

**Published:** 2021-06-22

**Authors:** Leslie B. Adams, Samuel L. K. Baxter, Alexandra F. Lightfoot, Nisha Gottfredson, Carol Golin, Leron C. Jackson, James Tabron, Giselle Corbie-Smith, Wizdom Powell

**Affiliations:** 1 Department of Health Behavior, Gillings School of Global Public Health, University of North Carolina at Chapel Hill, Chapel Hill, NC USA; 2 Department of Mental Health, Johns Hopkins Bloomberg School of Public Health, Baltimore, MD USA; 3 Department of Public Health Sciences, Clemson University, Clemson, SC USA; 4 Division of General Medicine and Epidemiology, Department of Medicine, UNC School of Medicine, University of North Carolina at Chapel Hill, Chapel Hill, NC USA; 5 Department of Social Medicine, UNC School of Medicine, University of North Carolina at Chapel Hill, Chapel Hill, NC USA; 6 Center for Health Equity Research, UNC School of Medicine, University of North Carolina at Chapel Hill, Chapel Hill, NC USA; 7 Health Disparities Institute, University of Connecticut, Hartford, CT USA

**Keywords:** Mental health, Depression, Black men, Concept mapping, Measurement

## Abstract

**Background:**

Despite cumulative socioeconomic disadvantage and risk factors, Black Americans have a lower prevalence of depression than whites. Given the emerging focus of depression as a public mental health crisis, culturally informed depression measures and scale development techniques are needed to better alleviate the mental health burden of socially marginalized populations. Yet, for Black men, race- and gender-related factors that position emotional vulnerability as a sign of weakness, may potentially mask the timely identification of mental health needs in this population. Thus, we address these gaps by employing a stakeholder-driven, community-engaged process for understanding Black men’s depression experience.

**Methods:**

We use concept mapping, a structured mixed methods approach, to determine how stakeholders of Black men’s health conceptualize their depressive symptoms. Thirty-six stakeholders participated in a three-phase concept mapping study conducted in 2018. Three separate stakeholder groups were engaged for this study, including Black men, Black women, and primary care providers.

**Results:**

Participants generated 68 characteristics of Black men’s depression which were reflected within five conceptual clusters: (1) physical states; (2) emotional states; (3) diminished drive; (4) internal conflicts; (5) communication with others; and (6) social pressures. Using a content analysis approach, we found that items comprising the “social pressures” cluster were not reflected in any common depression scales.

**Conclusions:**

Findings from this study illustrate the similar and divergent pathways in which Black men express depressed mood. Furthermore, concept mapping results also yield a novel opportunity for culturally informed scale development in future research.

**Supplementary Information:**

The online version contains supplementary material available at 10.1186/s12889-021-11137-5.

## Introduction

Major depressive disorder (MDD) is the most common mental health condition in the United States, impacting close to 17 million Americans [1, 2]. Previous research posits that depressive symptoms present uniformly across or within groups of individuals. However, emerging quantitative literature finds significant heterogeneity in depressive symptoms, particularly among racial and ethnic minorities [3]. Among Black Americans, depression is also a leading cause of morbidity and mortality, yet the rates of diagnosed major depressive disorder in the healthcare sector is lower than the general population [4]. Moreover, Black Americans experience unmet need for their mental health concerns, with only 25% of those in need of behavioral healthcare receiving treatment [1, 2]. Despite efforts to improve access to quality and culturally competent care, treatment initiation disparities for Black Americans persist [5, 6].

Despite the cumulative burden of social inequality and racial discrimination, both significant risk factors for mental health conditions, Black men have a lower prevalence of MDD than Black women and their male counterparts [2, 4]. Coupled with this paradox, Black men also experience prolonged severity and chronicity associated with depressive symptoms and fewer mental healthcare contacts than white men [7, 8]. Researchers have recently highlighted the divergence between lower depression diagnoses and rising rates of suicide completion among Black boys and men, which has necessitated national attention among researchers and policymakers [9–13]. Indeed, the notion that social environments influence depressive symptoms in Black communities has been amplified in light of research showing that the increased hypervisibility of police violence, discrimination, and killings of unarmed Black men lead to detrimental spillover effects in the mental health of this population [14–16]. However, the influence of these social inequities have been an underexplored aspect of research on Black men’s mental wellbeing, and ultimately, our understanding of how depressive symptoms are characterized in this population.

### Critiquing the status-quo of depression measurement

To date, researchers and clinicians have relied on validated measures and clinically-derived handbooks, such as the Diagnostic and Statistical Manual of Mental Disorders (DSM-V), to diagnose depression. However, studies assessing the reliability and validity of these measures in Black male populations have yielded mixed results [3, 17, 18]. A small, but robust, body of research finds that Black and male populations articulate depressive symptoms in ways that diverge from the DSM criteria. In a review by Sydney Hankerson and colleagues [19], they synthesized evidence on racial and gender biases that contribute to misdiagnosis of depression among African American men. Of note, they found studies that describe that African Americans with major depressive disorder (MDD) are more likely to somaticize their emotional problems and, thus, may result in fewer MDD diagnoses in the clinical setting. Researchers also reported that the language used to describe depression in focus groups comprised of African Americans diverged from DSM characterizations, such as irritability, negative thought processes, hopelessness, loneliness, and social isolation, among others [20, 21]. Additionally, existing measures to capture male depression (e.g. Gotland Male Depression Scale, Male Depression risk scales, etc.) were developed and validated in non-Black samples, which omits the complex interplay of racialized and gendered experiences that may manifest in Black men’s descriptions of depressive symptoms [22–24]. Collectively, these studies suggest that the DSM criteria of which most measures are derived may fail to identify (1) culturally nuanced symptoms and subsequently reduce opportunities for expanded set of symptom experiences as a function of the disorder and (2) how race- and gender-related factors intersect to inform Black men’s depression experience. Our study seeks to mitigate this gap using concept mapping, a participatory methodology [25].

### Description of the current study

Previously described limitations in depression measurement highlight the importance of engaging Black men directly to determine their depression experience. Concept mapping, a structured mixed methods approach to organize and operationalize stakeholders’ views on a particular research topic, is one such method that provides further evidence towards clarifying stakeholder-driven narratives about health [26]. The method combines qualitative data collection procedures and quantitative analytic approaches to create a pictorial representation of stakeholder inputs about a topic. In this study, we employed concept mapping to generate conceptualizations of how depressive symptoms were described in a community-based sample of Black men and their stakeholders. By employing a concept mapping approach to refine our understanding of the depression experience, Black men and their stakeholders are directly involved in the critical preliminary stages of scale refinement processes.

Our guiding hypothesis is that Black men’s marginalized social status in the United States fundamentally shapes their depression symptoms, and ultimately, the ways in which they conceptualize the depression experience. Specifically, we apply concept mapping to understand (1) the integration of race- and gender-related factors in Black men’s depression experience, and (2) the utility of concept mapping methodology to expand depression scale-refinement processes beyond existing DSM-V diagnostic criteria. By refining the conceptualization of depression with Black men and their stakeholders, we bridge important insights from extant research that posits that language around depression may diverge from DSM-V classifications of major depressive disorder [20, 27]. We assert that improved measurement of depression among Black men will provide more comprehensive opportunities for the early detection of depressive symptoms in community and clinical settings, thus mitigating the risk of morbidity and premature mortality associated with misdiagnosed or undetected symptoms. The goal of the study and our associated methodology is to provide a launch point for improved depression measurement, which may in turn, support better detection, diagnosis, and earlier entry points for Black men to seek mental healthcare.

## Methods

### Eligibility criteria and recruitment

Participants were eligible for this study if they were at least 18 years old, a current resident in the Research Triangle area of North Carolina, English-speaking, and self-identified as a Black man, Black woman, or primary care provider of any racial or ethnic background. Participants for the concept mapping process were recruited using a non-random purposive sample, which is the preferred sampling frame for concept mapping to achieve heterogeneity in the reflected items generated from participants [28]. Black men, women, and primary care providers were recruited to participate in different phases of the concept mapping study; Black men were involved throughout all three phases of the study, whereas Black women and providers were only involved in the initial brainstorming phase. This process was conducted in order to expand the pool of brainstormed items related to depression through the lens of key relational and therapeutic stakeholders in Black men’s health [27, 29, 30].

Black men and women stakeholder groups were recruited through targeted flyers distributed through community organizations and listservs of interest (e.g. fraternities, male-centered organizations, etc.), academic and community-based networks, such as gender-specific student organizations and male-centered mentoring organizations in predominantly Black neighborhoods, to generate a diverse range of eligible participants. Additional Black men were recruited using snowball sampling from previous participants. Primary care providers were recruited using direct contacts from Community Advisory Board (CAB) members and a recruitment services support team at University of North Carolina at Chapel Hill. Participants were compensated up to $50 for their participation in two in-person concept mapping sessions ($25 per session).

### Overview of concept mapping process

Data was collected from October 2017 to January 2018 in Durham, North Carolina in three phases: brainstorming; sorting; and interpretation. The lead author facilitated each phase and audio recorded sessions with stakeholders. The brainstorming phase consisted of group discussions during which participants were asked to brainstorm or “free list” ideas related to the focal question: “What are distinct characteristics of depression among Black men?” Two brainstorming sessions were conducted in-person with Black men and Black women, respectively. Primary care providers completed the brainstorming activity online due to time constraints. During the sorting phase, participants individually organized the list of brainstormed items into piles based on conceptual similarity. Participants labeled piles independently based on how they perceived the items related to each other. At baseline, participants completed a brief survey comprised of demographic information (e.g. age, occupation, etc.) and an assessment of depressive symptoms using the Center for Epidemiological Studies Depression (CES-D) 12-item scale [31]. The research protocol for our concept mapping study was approved by the Institutional Review Board at University of North Carolina at Chapel Hill and all study procedures were performed in accordance with relevant guidelines and regulations set forth by the institution. Informed written consent to participate in the study was obtained from all participants prior to the study initiation.

### Community advisory board overview

To maintain community perspectives throughout the study, we assembled an advisory board to guide the design, implementation, and analytic phases of our study. We convened a CAB of three members, two Black men and a Black woman primary care provider, who were able to offer a community-driven perspective of the research questions and process. CAB members were selected based on their familiarity with Black men’s health, ability to participate in periodic conference calls, interest in the research questions, and willingness to engage community leaders on the research topic. Recruitment of CAB members was conducted by convenience from community-based research studies focused on Black men’s health by research staff. Once the study launched, the CAB assisted with recruitment efforts, helped refine brainstormed items, and participated in the interpretation session at the conclusion of the study. CAB members were offered $60 for participation by the study’s primary investigator (first author).

## Analysis

**Fig. 1:**
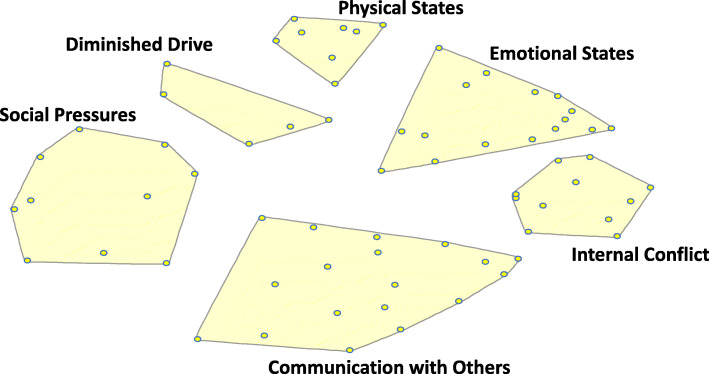
Six-cluster concept map of Black men’s depression characteristics (*n* = 36)

Data analysis was conducted using Concept Systems Global Max software. The software uses multidimensional scaling (MDS) and hierarchical cluster analysis (HCA) to yield a pictorial display which, for this study, focused on conceptualizations of Black men’s depression. MDS and HCA were used to derive a point map (see Supplementary Materials) and cluster map (Fig. 1). In the point map, points represent statements from the brainstormed activity. Points that were closer in proximity on the map indicated that participants often grouped these statements together during the sorting activity. A cluster map was generated from the point map using HCA with Ward’s algorithm to partition the points into non-overlapping cluster boundaries [28]. Clusters, defined by the content of the included points or statements with similar meaning and concept, indicated distinct concepts related to Black men’s depression. Concept Systems software generated a range (2–20) of potential clusters that were reduced to a meaningful number of clusters, labeled, and provided with operational definitions by the CAB and selected participants from previous phases of the study during the interpretation phase. During the interpretation phase, select members of the brainstorming and sorting phases came together with CAB members to validate study results and confirm the appropriate number of clusters that best represented conceptual domains of depression.

### Content analysis

We conducted a content analysis to compare concept map clusters to psychometric constructs identified through established factor analyses studies of several well-validated measures of depression. Namely, we compared our findings to the following measures: the 20-item Center for Epidemiologic Studies Depression Scale (CES-D) [31], the 9-item Patient Health Questionnaire (PHQ-9) [32], and the 21-item Beck’s Depression Inventory (BDI) [33]. Our content analysis compared each concept map cluster of the finalized concept map to confirmatory factor analysis results of the four selected depression scales. We analyzed both item wording and factor analyses for similarities in wording or phrasing of the construct. Concept mapping constructs were considered to be similar if equivalent content or wording was captured in the confirmatory factor analyses and resulting items in each depression scale.

## Results

This concept mapping study included 36 participants that represented three stakeholder groups: 23 Black men, 10 Black women, and 3 primary care providers. The brainstorming phase involved 21 participants: 8 Black men, 10 Black women, and 3 primary care providers. In total, the participating stakeholder groups yielded 119 non-unique statement items. The CAB removed redundant statements and created items that could be easily discernible by participants in the sorting phase of the study. This resulted in 68 unique statements that were adequately responsive to the focal question (listed in Table 1). The sorting phase consisted of 22 Black men who comprised the analytic sample for study results. Black men in the analytic sample were predominantly 18–29 years old (*n* = 11, 50%), single (*n* = 15, 68%), and employed either full or part-time (*n* = 14, 64%). The interpretation session included five participants representing all stakeholder groups, including the three members of the CAB.

**Table 1:** Cluster Names and Brainstormed Statements

Cluster Name	Statement (# represents position on point map)
**Physical States**	High Blood Pressure (1)
	Self-harm or suicidal behavior (11)
	Binge eating (12)
	Insomnia (37)
	Over-sleeping/Sleeping frequently (38)
	Heart palpitations (48)
	Weight loss (49)
	Weight gain (50)
**Diminished Drive**	Untidy or messy appearance (e.g. home, personal hygiene, etc.) (3)
	Not able to complete tasks (5)
	Not being able to provide for your family (13)
	Not able to complete life goals (e.g. finish school, get a job, travel etc.) (17)
	Excessive substance use (e.g. marijuana, cigarettes, alcohol, etc.) (60)
**Social Pressures**	Not being able to keep up appearances (e.g. different clothes, shoes, etc. than peers) (14)
	Fear of the unknown consequences of today’s political environment (22)
	Adherence to norms of competition with other men (e.g. “I have to do better than the other guy”) (23)
	Adherence to cultural norms of success and power (e.g. Keeping up with the Joneses, having a large salary, having a high-power job, etc.) (26)
	Constant strain to “do what you have to do to survive” (e.g. support kids, pay bills, etc.) (29)
	Lack of work/life balance (33)
	Ignoring physical symptoms (e.g. injuries, pain, discomfort, etc.) (46)
	Excessive engagement in activities that improve outward appearance (e.g. over exercise, overspending, etc.) (61)
	Seeking happiness through accumulated materials (e.g. clothes, shoes, etc.) (62)
	Increased attendance at religious institutions (e.g. church, mosque, etc.) (66)
**Communication with Others**	Unable to communicate properly (e.g. delayed or ignored emails, calls texts, etc.) (2)
	Isolation from others (e.g. friends, family, romantic partners, etc.) (7)
	Discouraging others (“misery loves company”) (9)
	Unclear or limited communication (e.g. short verbal responses, unclear body language, etc.) (16)
	Feeling hopeless due to prolonged exposure to state violence against Black people (e.g. police shootings on social media, news coverage, etc.) (20)
	Blunted emotional expression or flat affect (e.g. not crying, no facial changes, etc.) (31)
	Withdrawal from everyday activities (e.g. hobbies, interests, etc.) (34)
	Not able to maintain romantic relationships (41)
	Stuffing down emotions (42)
	Ignoring support from others (45)
	Personality/Mood changes in romantic relationships (51)
	Volatile behavior towards others (52)
	Sharing emotions through indirect forms of communication (e.g. writing long, emotional status updates on social media, letters, emails, etc.) (57)
	Blaming others for issues (58)
	Only reflecting or reminiscing on certain life stages (e.g. adolescence, early adulthood, etc.) (59)
	Change in sexual behavior in relationships (e.g. loss of interest, aggression, etc.) (63)
	Reestablishing relationships with past acquaintances (e.g. friends, romantic partners, etc.) (64)
	Seeking closure with others/wrapping up loose ends (65)
**Emotional States**	Not able to “get up and go” (4)
	Lack of motivation (6)
	Anger (10)
	Feeling “sick” (15)
	Low self-esteem (24)
	Being stagnant or stuck in life (25)
	Laziness (28)
	Feeling out of control (32)
	Feeling irritated or agitated (35)
	Feeling fatigued (36)
	Feeling hopeless (39)
	Feeling frustrated (43)
	Bursts of crying (44)
	Not feeling like yourself (47)
	Worry (55)
	Anxiety (67)
**Internal Conflict**	Having a pessimistic outlook or negative mindset (8)
	Feeling guilty (18)
	Feeling unqualified (e.g. imposter syndrome) (19)
	Having a heightened sense of fear or dread (21)
	Not feeling supported by the Black community (27)
	Not feeling valued for your work/Not seeing the benefits of hard work over time (30)
	Feeling like things are “off” (40)
	Feeling attacked/Defensiveness (53)
	Change in mood over time as men age (54)
	Feeling helpless due to the aging process (e.g. increased reliance on assistance from others) (56)
	Numbness, melancholy, or lack of engagement that can be observed by others (e.g. “There’s no joy in your eyes”) (68)

### Cluster map

Figure 1 illustrates the final cluster map derived from data collection during the study’s sorting phase. The final cluster map consists of six conceptual clusters labeled as (1) Physical States; (2) Emotional States; (3) Internal Conflict; (4) Communication with Others; (5) Social Pressures; and (6) Diminished Drive. Each cluster label represents the common theme of the statements in that particular cluster and was named by study participants during the interpretation phase. Each point on the cluster map corresponds to the statements reflected in Table 1.

### Description of depression clusters

The “physical states” cluster is an eight-item cluster that includes statements describing physical manifestations of negative affect. Statements in this cluster included somatic symptoms or clinical diagnoses, such as *high blood pressure*, *heart palpitations, binge eating, and insomnia*. “Emotional States” is a 16-item cluster that describes both the internalizing and externalizing aspects associated with depressed mood, such as *anger* and *feeling hopeless*, and included statements related to feeling *stagnant, fatigued, or frustrated*. The “communication with others” cluster consists of 18 brainstormed statements that describe challenges of maintaining interpersonal communication across multiple relationship domains, including family, friends, and romantic partners, as Black men deal with depressive symptoms. Statements in this cluster included being *unable to communicate properly with others*, *isolated from others*, and having *unclear or limited communication*. One participant described statements in this cluster as “what people withhold” when dealing with depressive symptoms. Of note, this cluster not only included verbal communication, but also elements of non-verbal communication that may be emotionally driven, such as *limited facial expression or blunted affect*. The “social pressures” *cluster included* ten items related to the strains associated with achieving traditional male gender roles. Statements in this cluster included *adherence to norms of competition with other men* and *maintaining notions of success and power*. The “internal conflict” cluster consisted of 11 brainstormed statements that named internalized sentiments such as *feeling “off”* and *pessimistic.* Notably, this cluster also includes items related to internalized standings in the Black community (e.g. not feeling supported in the Black community). Finally, “diminished drive” includes five brainstormed statements related to the *unfinished business* and *untapped potential* of Black men. Statements in this cluster included *not able to complete tasks* and *not able to complete life goals, such as school and employment*. Male participants continually highlighted gender norms related to *not being able to provide for your family* throughout the brainstorming phase as a key catalyst of depressive symptoms for Black men.

### Comparison of concept mapping clusters and existing depression scales

Table 2 illustrates results from our content analysis that compared concept mapping results with items and constructs identified in existing depression scales. We found that none of the existing measures included in our content analysis describe all of the clusters generated by our concept mapping study. Notably, all but one cluster, “social pressures”, was reflected in one or more of the four depression scales. Items and concepts related to “physical states” and “emotional states” as well as “diminished drive” clusters were described in factor analyses studies for each of the four scales.

**Table 2:** Comparison of Concept Mapping Clusters with Commonly-Used Psychometric Assessments for Depression

	Concept Mapping Clusters						Items
	Physical States	Emotional States	Internal Conflict	Communication with Others	Social Pressures	Diminished Drive	
Center for Epidemiological Studies Depression Scale (CES-D)	X	X	–	X	–	X	20
Patient Health Questionnaire (PHQ9)	X	X	–	–	–	X	9
Beck’s Depression Inventory (BDI)	X	X	X	–	–	X	21

X: Construct reflected in depression measure (row)

--: Construct absent in depression measure (row)

## Discussion

The purpose of this study was to understand Black men’s unique conceptualizations of depression and the parallels and divergence from existing depression scales. The concept map we developed depicts six distinct clusters that characterize depression among Black men and provides novel insights towards Black men’s unique depression experience. As hypothesized, results from the concept mapping study highlight domains of depressive symptoms in Black men that are not currently captured in current depression scales. Our results add additional support to evidence indicating associations between somatic depressive symptoms and self-management behaviors among men. Additionally, our study extends evidence on the extent to which Black men’s gender role strain may serve as a key indicator of depressed mood [20, 34–36].

The “physical states”, “emotional states”, and “diminished drive” clusters were captured in factor analysis studies of the CES-D, PHQ-9, and BDI measures. However, some key differences were identified in our content analysis. Notably, the “social pressures” cluster identified items that were not reflected in any commonly used measures of depression. This cluster consisted of a diverse range of items that broadly reflected the impact of the social environment on Black men’s sense of self (e.g., *competitive drive, adherence to gender norms of success and power*, etc.). Overall, this cluster indicated that the tensions men experienced trying to meet societal gender role expectations were a key aspect of Black men's experience of depression in their daily lives. The *“*internal conflict” cluster also identified items that were not reflected broadly across commonly used measures of depression. Items within this cluster highlight the cumulative burden (e.g., no joy in their eyes) and undisclosed feelings (e.g., feeling unqualified, not valued for your work, and attacked) that may result from Black men enduring social inequities.

Moreover, items from the PHQ-9 scale mirrored only three out of the six concept mapping clusters (“physical states”, “emotional states”, and “diminished drive”). One potential rationale for this result may be due, in part, to the fewer number of items that comprise the PHQ-9 measure, compared to the other instruments (20 items or more) and brainstormed items from our study (68). Moreover, CES-D factor analyses studies identify interpersonal conflict as a common domain of the measure, typically comprised of two items related to “people dislike me” and “people were unfriendly” [31]. Our study found that the “communication with others” cluster shares both similarities and key divergences from items that comprise the interpersonal conflict domain of the CES-D [31]. For instance, our “communication with others” domain expands to include interpersonal challenges that may manifest among Black men living with depression, such as disruptions in in-person, calls, texts, or social media presence. Additionally, in contrast to the two items that comprise the CES-D’s interpersonal domain, our cluster consisted of 18 items, which indicates that more expansive conceptualizations of interpersonal disruptions, relational ties, and peer relationships are warranted in the study of Black men’s depression. A recent study by Adams and colleagues (2018) [17], confirms, through a CES-D factor analyses, that the interpersonal conflict and negative affect features of depression may be intrinsically linked for Black men and connect to our current findings to explain the size and variation of the “communication with others” cluster. Forthcoming research should further explore the interplay between social and psychological disruptions in Black men’s everyday experience as a possible signal of depressive symptomatology.

Although these cluster domains were widely represented across survey instruments, the scope of items reflected in each domain was not comparable. For example, CES-D factor analysis studies identify somatic symptoms that are similar to items in our “physical states” cluster. However, CES-D items do not include the same type of cardiac-related items that are present in our concept mapping domain (e.g. heart palpitations and high blood pressure). Similarly, the Beck Depression Inventory (BDI) includes items related to experiencing guilt and feeling worthless that were mirrored in the “internal conflict” [33]. However, our domain also includes items that reflect conflicts in within-group social cohesion (e.g. not feeling supported by the Black community) and life course-related transitions (e.g. change in mood over time as men age). Taken together, there may be additional physical and psychological manifestations of depressed mood that clinicians should probe with Black male patients.

There are some limitations that impact the generalizability of our findings. The sample was derived from purposive sampling methods. This sampling technique is consistent with previous sampling frameworks used in preceding concept mapping studies, which seek to produce a subjective conceptual framework based on participant responses [26, 28]. The sample consisted of Black men residing in a metropolitan area of the southeastern region of the United States so our findingsmay be limited to a Black men residing in similar spatial and political contexts.

Despite these limitations, concept mapping methodology provided a valuable tool to yield stakeholder-driven perspectives on depression among Black men. This method was particularly advantageous for engaging a broad range of stakeholders and provides a foundation for the refinement of existing depression scales and the development of new measures to capture the full range of depressive symptom presentation among Black men. Furthermore, our study design prioritized the perspectives of Black men, a population seldom engaged in health research [37]. Centering Black men to achieve our research aims validates their lived experiences and provides a foundation to guide future action-oriented research to support Black men’s mental wellbeing in both community and psychiatric settings.

## Public health implications

Our study provides a critical first step in refining measurements of depressive symptoms among Black men, a highly underserved population in both psychometric and public health research. Specifically, our concept mapping results serve as the initial blueprint for forthcoming scale development research to better measure Black men’s depressive symptoms in community and clinical settings. Future studies guided by our brainstormed items and cluster map will explore the development of a multi-dimensional depression measure, which would include item refinement, cognitive testing, and the evaluation of construct and predictive validity in a fully-powered sample of Black men [38]. The conceptual domains derived from this study may also guide hypothesis testing in validation studies that compare study results with existing general and gender-specific scales, such as the Center for Epidemiologic Depression Scale, Gotland Male Depression Scale, and the Black Men’s Experiences Scale [23, 31, 39]. Collectively, future research stemming from our concept mapping study will catalyze innovative strategies to detect and mitigate Black men’s depressive symptoms at earlier timepoints.

The findings from this study also have implications for how healthcare providers can better serve Black men. Primary care settings are the most accessible point of care for many Black men. Thus, clinicians should ensure stronger therapeutic alliances with their Black male primary care patients. Depressed Black men may be more likely to present with medically unclear somatization, which is consistent with previous literature on depression recognition in the healthcare setting [40, 41]. Consequently, special attention should also be paid to how Black men articulate, during patient-provider interactions, aspects of their everyday lives and social strain to improve stronger therapeutic linkages and recognition of depressive symptoms in the primary care setting. Previous studies examining this phenomenon found that Black men’s depressive symptoms may be articulated in ways that diverge from hallmark signs of depressed mood due to prevailing masculine scripts that disparage crying, vulnerability, and help-seeking [20, 21, 27, 29].

Given these findings, we argue that current depression scales may not capture the full range of depressive symptom expression among Black men. Currently, many depression measures used in the clinical setting are skewed towards the presence of negative affect or depressed mood. Yet, this study shows that Black men may portray symptoms in the healthcare setting that are more nuanced, such as somatization and relational disruptions. The omission of these subtler descriptors in common depression scales may explain the low prevalence estimates of Black men’s depression in population-based studies compared to the general population [40]. Misdiagnosis, underreporting, or delayed treatment of depression in Black men may have potentially deleterious outcomes in this population, including physical health comorbidities, psychiatric hospitalization, or death by suicide [1, 20, 36]. Consequently, researchers working to improve Black male life expectancies must consider intervening on the diverse range of symptoms that influences observed mental health disparities in this population.

## Supplementary Information

**Additional file 1.**

## Data Availability

The dataset generated and analyzed for this study is available through the Primary Investigator (LBA) on reasonable request.
