# Supplementary figures and images for: Refining Black men’s depression measurement using participatory approaches: a concept mapping study
Source: BMC Public Health. 2021 Jun 22;21:1194. doi: 10.1186/s12889-021-11137-5 (PMC8220674; doi:10.1186/s12889-021-11137-5)

**Appendix A: Point Map of Black men’s depression characteristics (n=36)**


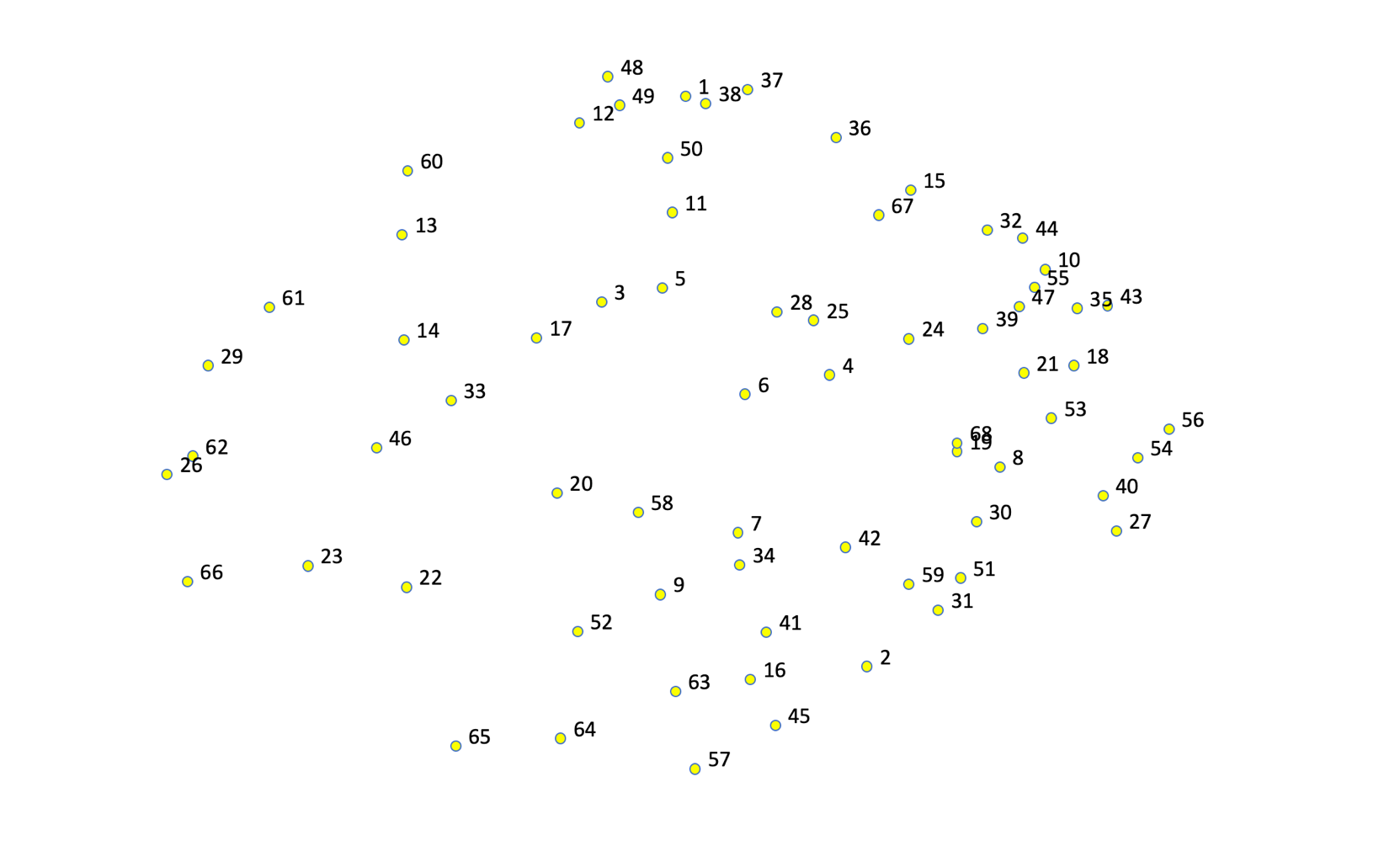

Supplement: Supplementary file 1 — Additional file 1. [file 12889_2021_11137_MOESM1_ESM.docx]
